# Protective Effects of Resveratrol Against Amyloid Beta‐Induced Toxicity in Neuroblastoma Cells: Insights from Cell Viability and cellular energetics.

**DOI:** 10.1002/alz70859_102456

**Published:** 2025-12-25

**Authors:** Manas Chakraborty, Veer Gupta

**Affiliations:** ^1^ Deakin University, Waurn Ponds, VIC Australia

## Abstract

**Background:**

Effective treatments for early cognitive impairment and Alzheimer’s disease (AD) remain limited. Given the multifactorial nature of AD, therapeutic strategies must focus on targeting disease‐specific biochemical pathways. Metabolic disruptions play a critical role in cognitive decline, prompting research into alternative disease modifiers like phytochemicals. This study investigated the therapeutic potential of resveratrol, a biologically active terpenoid derived from *Ginkgo biloba*, to alleviate amyloid‐induced cellular dysfunction. Known for its antioxidant and anti‐inflammatory properties, resveratrol may modulate oxidative stress and inflammation, key contributors to neurodegeneration.

**Method:**

The study utilized the BE(2)‐M17 neuroblastoma cell line to model AD. Cells were differentiated into mature neurons using 10 µM retinoic acid and subsequently treated with 20 µM amyloid beta (Aβ) for 24 hours. Prior to Aβ exposure, cells were pre‐treated with varying concentrations of resveratrol (1 µM, 5 µM, 10 µM, 15 µM, and 20 µM) for 24 hours. Three assays—CellTiter‐Glo, ROS, and MTT—were performed to assess resveratrol’s effects on cell viability, oxidative stress, and mitochondrial function.

**Result:**

The CellTiter‐Glo assay revealed that pretreatment with 10 µM resveratrol resulted in the highest ATP production and cell viability (*p* = 0.0386), indicating improved cellular energy status and survival under Aβ exposure. The ROS assay demonstrated that 10 µM resveratrol significantly reduced oxidative stress induced by Aβ (*p* = 0.049), highlighting its ability to counteract a major driver of neurodegeneration. Lastly, the MTT assay showed that 10 µM resveratrol effectively mitigated Aβ‐induced mitochondrial dysfunction (*p* = 0.013), emphasizing its protective impact on mitochondrial integrity.

**Conclusion:**

Resveratrol at 10 µM emerged as the most effective concentration across all assays, demonstrating its ability to enhance cell viability, reduce oxidative stress, and restore mitochondrial function. These findings suggest that resveratrol’s modulation of energy metabolism and cellular homeostasis holds promise as a therapeutic approach for Alzheimer’s disease.

